# Association of Noise Exposure, Perceived Noise Impact, and BMI on Nonalcoholic Fatty Liver Disease Among Chinese Seafarers: Nationwide Population-Based Survey

**DOI:** 10.2196/57844

**Published:** 2026-10-13

**Authors:** Ruizi Shi, Shuai Zhou, Zhitao Yang, Erzhen Chen, Hanbing Shang, Ying Zhou

**Affiliations:** 1 Shanghai Research Institute of Aviation Medicine, Ruijin Hospital, Shanghai Jiao Tong University School of Medicine, No.197 Ruijin Er road, Shanghai, 64370045, China, 86 600603 ext 64370045; 2 Emergency & Critical Care Medicine Center, Ruijin Hospital, Shanghai Jiao Tong University School of Medicine, Shanghai, China; 3 Division of Medical Affairs, Ruijin Hospital, Shanghai Jiao Tong University School of Medicine, Shanghai, China; 4 Department of Emergency, Ruijin Hospital, Shanghai Jiao Tong University School of Medicine, Shanghai, China; 5 Department of Neurosurgery, Ruijin Hospital, Shanghai Jiao Tong University School of Medicine, Shanghai, China

**Keywords:** noise exposure, perceived noise impact, nonalcoholic fatty liver disease, NAFLD, seafarers, occupational health, BMI

## Abstract

**Background:**

Nonalcoholic fatty liver disease (NAFLD) is a global public health challenge, yet its relationship between occupational noise exposure and perceived noise impact remains unclear.

**Objective:**

We characterized maritime noise exposure patterns and examined the associations of noise exposure, perceived noise impact, and BMI with NAFLD among seafarers.

**Methods:**

A population-based survey was conducted from March to May 2024 through the China Shipowners’ Association. Participants completed a structured online questionnaire. Noise exposure was assessed via self-report, and NAFLD presence was determined by self-reported physician diagnoses based on the 2018 updated NAFLD guidelines.

**Results:**

A total of 8143 male seafarers was included, with an overall NAFLD prevalence of 18.5% (n=1503). The prevalence was significantly higher among seafarers with a BMI of 24.0 kg/m² or above (25.9%, n=1261), chronic noise exposure (19.4%, n=1293), and higher perceived noise impact (slightly: 16.9%, n=589; moderate: 19.1%, n=359; significant: 22.5%, n=511). Among seafarers with a BMI of 24.0 kg/m² or above, chronic noise exposure and higher perceived noise impact were significantly associated with an elevated risk of NAFLD. After full adjustment, the combined association of chronic noise exposure and higher perceived noise impact remained significantly associated with increased NAFLD risk, with consistent patterns observed across both workplace and rest area settings. A significant interaction was also observed between rest-area noise exposure and perceived noise impact in this BMI subgroup.

**Conclusions:**

Noise exposure, perceived noise impact, and BMI were significantly associated with NAFLD among seafarers. These findings highlight the importance of optimizing occupational health benefits, especially among those with metabolic disorders.

## Introduction

Nonalcoholic fatty liver disease (NAFLD) has emerged as a critical global public health challenge among chronic noncommunicable diseases [1]. Over the past decades, its worldwide prevalence has risen dramatically, from 25.5% before 2005 to 37.8% after 2016 [2]; in China, the prevalence has also increased significantly, climbing from 23.8% in the early 2000s to 32.9% in 2018 [3]. NAFLD encompasses a spectrum of liver conditions, progressing from simple hepatic steatosis to steatohepatitis, which may gradually develop into fibrosis and ultimately advance to cirrhosis or hepatocellular carcinoma [4]. Furthermore, NAFLD is associated with various extrahepatic complications, including cardiovascular diseases, type 2 diabetes, and chronic kidney disease [5]. Its pathogenesis involves multiple factors, such as genetic, nutritional, and environmental determinants [6], with chronic noise exposure and its related psychological impacts emerging as a potential environmental trigger that has received insufficient attention.

Chronic noise exposure is known to significantly elevate cortisol levels and contribute to obesity-related metabolic disorders [7,8]—2 key risk factors for NAFLD. Animal experiments demonstrate that 3 months of chronic low-intensity noise exposure exacerbates NAFLD in high-fat diet–fed mice by activating the hypothalamic-pituitary-adrenal (HPA) axis [9]. Additionally, noise may indirectly promote hepatic lipid accumulation by disrupting circadian sleep rhythms [10]. This evidence is particularly relevant to seafarers, a globally neglected occupational group who experience unavoidable noise exposure during voyages alongside a high metabolic burden. The maritime settings are plagued by noise pollution from propulsion systems, power plants, and structural vibrations aboard vessels [11], and seafarers are continuously exposed to excessive noise during voyages, as both working places and living quarters are confined to ships. Notably, obesity-related metabolic disorders are already a major health burden among seafarers [12], further highlighting their vulnerability to NAFLD.

However, few studies have explored the unique exposures among seafarers that are associated with NAFLD. Most studies on NAFLD focus on traditional metabolic factors (eg, BMI, diet), which are typically derived from the general population, while 2 important factors—chronic noise exposure and perceived noise impact—remain understudied in the context of NAFLD. Crucially, emerging evidence indicates that perceived noise and its perceived impact, as the key sources of psychological distress, may independently amplify some disease risks [13-15]. However, such effects have been predominantly documented in mental health outcomes, with no exploration of their potential link to NAFLD.

Therefore, even studies investigating the association between noise exposure, perceived noise impact, and NAFLD remain scarce in the general population, let alone in occupational groups such as seafarers. To the best of our knowledge, no comprehensive epidemiological studies have systematically evaluated the prevalence of NAFLD or its association with noise exposure or perceived noise impact specifically among seafarers. Given the potential progression of NAFLD to liver fibrosis, it poses long-term health and occupational risks for seafarers, and seafarer health directly impacts workforce efficiency and maritime safety, as poor health may impair job performance and increase accident risks [16]. Since NAFLD is reversible, investigating its disease burden among seafarers and analyzing the impacts of noise exposure, noise sensitivity, and metabolic disorder are essential for targeted prevention strategies.

Against this backdrop, we conducted a large-scale cross-sectional study with three primary objectives: (1) to estimate the prevalence of NAFLD among seafarers; (2) to characterize the unique noise exposure patterns in maritime environments; and (3) to examine the association of noise exposure, perceived noise impact, and BMI on NAFLD. This study may yield evidence not only for the specific prevention and treatment of NAFLD but also for an evidence base to inform ship design and health management strategies.

## Methods

### Study Design and Participants

This cross-sectional study is the baseline survey of the Seafarers Health Cohort of China, which is an ongoing large-scale health surveillance cohort aiming to promote physical and mental health among seafarers in China.

Participants were recruited from March to May 2024 in collaboration with the China Shipowners’ Association (CSA). As a national authoritative maritime industry organization, the CSA consists of nearly 400 domestic and international member entities, including shipowners, shipping operators, vessel managers, as well as shipping insurance, financial institutions, maritime research institutes, and universities. Its member enterprises account for more than 85% of China’s national maritime transport capacity. This membership system encompasses both national leading shipping conglomerates and numerous local professional maritime service institutions.

Considering seafarers’ frequent cross-regional mobility and irregular work schedules, all recruitment and data collection procedures were conducted online using a structured electronic questionnaire hosted on the Wenjuanxing survey platform [17]. This allowed participants to complete the survey either on board or on shore leave, without geographical constraints. A standardized, multistage recruitment procedure was followed across all participating institutions. The research team developed a unified study protocol and the electronic questionnaire. The questionnaire link and recruitment notice were distributed by the CSA through its official communication channels to the designated contacts at each participating enterprise. The coordinators were then responsible for forwarding the survey invitation to all active seafarers within their respective companies. The recruitment strategy covered all occupational ranks and departments, with no artificial screening or selective exclusion. All eligible seafarers accessed the survey voluntarily and completed it independently, free from managerial supervision or external interference. Participants were informed of their right to decline or withdraw from the survey at any time.

Several exclusion criteria were applied to the final analytical sample. Participants with preexisting hepatic conditions were excluded, including viral hepatitis (hepatitis B, C, etc), drug-induced liver injury, autoimmune liver diseases, and inherited metabolic liver disorders. Female seafarers accounted for merely 0.85% (n=70) of the original sample and were excluded from formal analyses due to extremely limited sample size. The characteristic comparisons between excluded female seafarers and included male seafarers are presented in Table S2 (Multimedia Appendix 1). Additionally, participants with missing BMI records were also excluded (Figure S1 in Multimedia Appendix 1).

### Ethical Considerations

This study was approved by the Institutional Review Board of Ruijin Hospital (approval number: 2023248). Written informed consent was obtained from all study participants before enrollment. Prior to data analyses, complete anonymization of all study datasets was performed to protect participants’ privacy and maintain information confidentiality. No financial remuneration was offered to participants for their involvement in this research. In addition, none of the images and figures in the manuscript and supplementary materials contains any information that could identify individual participants.

### Measurement

#### Noise Exposure

Noise exposure among seafarers was assessed using a self-designed 4-item scale, developed based on previous studies and expert consultations [15,18]. The first question assessed whether seafarers had chronic noise exposure, such as crew working in the engine department. The second and third questions required seafarers to rate the noise levels in their working environment and rest area, respectively, using the 4-point Likert scale: quiet, moderate, noisy, and very noisy. For subsequent analyses, to avoid sparse strata with insufficient sample sizes, we combined quiet with moderate and noisy with very noisy, yielding 2 categories: quiet or moderate and rather noisy. Finally, the fourth item evaluated the perceived impact of noise exposure on rest and sleep during the voyage, also scored on a 4-point Likert scale: no effect, slight, moderate, and significant.

#### BMI Category

The BMI was calculated by dividing weight in kilograms by the square of height in meters [BMI = weight (kg)/height (m) [2]. In accordance with the Chinese health industry standard *Criteria for weight classification of adults* (WS/T 428‐2013), all participants were stratified into 2 subgroups: normal weight (BMI <24.0 kg/m²) and overweight or obese group (BMI ≥24.0 kg/m²) [19].

#### Measurement of Covariates

Covariates in this study included demographic features, lifestyle, occupational characteristics, and comorbidity. Demographic variables included age, ethnicity (Han vs ethnic minority), household registration type (Hukou; rural vs urban), marital status (unmarried, married, or others), and education level (bachelor’s degree or higher vs below). Lifestyle variables covered smoking status (never, previous, or current), alcohol drinking frequency, physical activity habits both onshore and offshore, dietary patterns, and sleep duration. Alcohol drinking behavior was evaluated based on drinking frequency in the past month, categorized as 4 or more times/week, 1‐3 times/week, 1‐2 times/month, and less than 1 time/month. Participants who reported drinking alcohol 4 or more times/week were defined as frequent drinkers. In addition, daily ethanol intake (grams) was further calculated for each participant using the formula: Alcohol intake (g) = consumed volume (mL) × ethanol concentration (%) × 0.79 (density conversion coefficient of ethanol). Dietary patterns were constructed based on 3 items: frequency of breakfast consumption, regularity of meal times, and balance of meat-to-vegetable intake in daily meals. A “healthy diet” was defined as meeting all 3 of the following criteria: consuming breakfast 5 or more times per week, having meals at regular times 5 or more times per week, and maintaining a balanced mix of meat and vegetables in the daily diet. Occupational characteristics included years of service in the current position, months per shipboard duty tour, daily working hours on broad, number of job position changes, shift work status (yes vs no; for those who worked shifts, periods were categorized as 4:00 AM-8:00 AM or 4:00 PM-8:00 PM, 8:00 AM-12:00 PM or 8:00 PM-12:00 AM, 12:00 AM-4:00 AM or 12:00 PM-4:00 PM, and others), and monthly income (>8000 vs ≤8000 yuan [CNY ¥1=US $0.149 as of September 9, 2026]). Comorbidities included hypertension, dyslipidemia, and diabetes mellitus/impaired glucose tolerance (DM/IGT).

#### Assessment of Outcome

NAFLD status was ascertained using self-reported physician-diagnosed fatty liver disease data obtained from the study questionnaire, which allowed assessment of the disease burden among maritime occupational workers. In accordance with the *2018 updated guidelines for NAFLD prevention and management* [20], individuals with excessive alcohol consumption (defined as ≥30 g/day for men) were excluded from the NAFLD case definition. Those with a positive fatty liver diagnosis and alcohol intake below this threshold were classified as having NAFLD.

### Statistical Analysis

Descriptive characteristics of seafarers were described as mean (SD) or median (IQR) for continuous variables and numbers and percentages for categorical variables. The Student *t* test and Kruskal-Wallis tests were used for continuous variables, and *χ*^2^ test was used for categorical variables in comparing characteristics differences between the groups, as appropriate. Harman’s single-factor test incorporating all 123 questionnaire items was conducted to assess common method bias. The first factor accounted for 19.94% of the total variance, which fell below the 40% threshold, indicating that common method bias was unlikely to be a major concern (Table S1 in Multimedia Appendix 1).

The prevalence of NAFLD was estimated across strata by demographic characteristics, lifestyle factors, occupational characteristics, comorbidity, noise exposure, as well as perceived impact of noise. To explore the associations of noise exposure and perceived noise impact with NAFLD, we performed a series of age-adjusted and fully adjusted logistic regression, calculating odds ratios (ORs) and 95% CIs. Next, we estimated the prevalence, ORs, and 95% CIs of NAFLD stratified by noise exposure and BMI, as well as by perceived noise impact and BMI. In addition, we explored the combined associations of noise exposure and perceived noise impact with NAFLD and further examined these combined associations stratified by noise levels at different locations (workplace and rest areas). Multiplicative interactions between noise exposure, location-specific noise levels, and perceived noise impact were tested by including product terms in the regression models, and the corresponding interaction *P* values were calculated. All models were adjusted for age, educational level, marital status, household registration, smoking status, physical activity, dietary habits, shift work, monthly income, sleep duration, hypertension, dyslipidemia, DM/IGT, and BMI as a continuous variable.

To assess the robustness of our findings, we conducted sensitivity analyses excluding participants with hearing loss and reestimated both the independent and combined associations. All analyses were performed using Stata (Stata Corporation). A 2-tailed *P* value of <.05 was considered to be statistically significant.

## Results

### Characteristics of Chinese Seafarers

A total of 8143 male seafarers were included in the final analysis, with a median age of 39 (IQR 33‐49) years. Over half (n=4868, 59.8%) of the participants had a BMI of 24.0 kg/m^2^ or above. The demographic characteristics of the study population are presented in Table 1. Compared with those without NAFLD, individuals with NAFLD tended to be older and more likely to be married, and had higher proportions of smoking, frequent alcohol consumption, and lack of regular exercise. They also tended to have shorter sleep duration. Participants with NAFLD also had significantly higher prevalence of hypertension, dyslipidemia, and DM/IGT (all *P*<.001). Notably, the proportions of overweight or obesity, exposure to chronic workplace noise, and high perceived noise impact were significantly higher among individuals with NAFLD. The distributions of various noise exposure types and perceived noise impact are illustrated in Figures S2 to S4 (Multimedia Appendix 1).

**Table 1.** Demographic characteristics of Chinese seafarers.

Variables	Total (N=8143)	Non-NAFLD^a^ (n=6640)	NAFLD (n=1503)	*P* value
BMI ≥24.0 (kg/m^2^), n (%)	4868 (59.8)	3602 (54.3)	1266 (84.2)	<.001
Chronic noise exposure, n (%)	6670(81.9)	5372 (80.9)	1298 (86.4)	<.001
Perceived noise impact, n (%)				<.001
No effect	501 (6.2)	463 (7.0)	38 (2.5)	
Slightly	3490 (42.9)	2899 (43.6)	591 (39.3)	
Moderate	1879 (23.1)	1520 (22.9)	359 (23.9)	
Significant	2273 (27.9)	1758 (26.5)	515 (34.2)	
Age groups^b^ (y), n (%)				<.001
<30	1532 (18.9)	1383 (20.9)	149 (9.9)	
30-<40	2891 (35.6)	2397 (36.2)	494 (33.0)	
40-<50	1883 (23.2)	1467 (22.2)	414 (27.6)	
≥50	1813 (22.3)	1372 (20.7)	441 (29.4)	
Ethnicity, n (%)				.09
Minor	281 (3.5)	240 (3.6)	41 (2.7)	
Han	7862 (96.5)	6400 (96.4)	1462 (97.3)	
Hukou, n (%)				<.001
Rural	4254 (52.2)	3681 (55.4)	573 (38.1)	
Urban	3889 (47.8)	2959 (44.6)	930 (61.9)	
Marital status, n (%)				<.001
Unmarried	1840 (22.6)	1642 (24.7)	198 (13.2)	<.001
Married	6086 (74.7)	4834 (72.8)	1252 (83.3)	
Others	217 (2.7)	164 (2.5)	53 (3.5)	
Bachelor’s degree or higher, n (%)	1669 (20.5)	1326 (20.0)	343 (22.8)	.01
Smoking status, n (%)				<.001
Never	3973 (48.8)	3319 (50.0)	654 (43.5)	
Previous	2489 (30.6)	1957 (29.5)	532 (35.4)	
Current	1681 (20.6)	1364 (20.5)	317 (21.1)	
Often drinking, n (%)	222 (2.7)	167 (2.52)	55 (3.66)	.01
Daily alcohol intake, n (%)	0 (0‐0.68)	1367 (20.6)	314 (21)	
Exercise habits, n (%)				<.001
No regular exercise routine	4894 (60.1)	3937 (59.3)	957 (63.7)	
Frequent exercise on board	368 (4.5)	295 (4.4)	73 (4.9)	
Frequent exercise at home	1856 (22.8)	1526 (23.0)	330 (22.0)	
Frequent exercise both	1025 (12.6)	882 (13.3)	143 (9.5)	
Healthy diet, n (%)	5065 (62.2)	4161 (62.7)	904 (60.1)	.07
Times of job position changes, n (%)				.03
0	6466 (79.4)	5308 (79.9)	1158 (77.0)	
1	1291 (15.9)	1030 (15.5)	261 (17.4)	
2	386 (4.7)	302 (4.6)	84 (5.6)	
Years on current job, median (IQR)	4 (2‐10)	4 (2‐10)	2 (5‐12)	<.001
Months per shipboard duty, mean (SD)	7.6 (1.5)	7.7 (1.4)	7.6 (1.6)	.007
Daily work hours on a ship, mean (SD)	8.7 (1.4)	8.6 (1.4)	8.9 (1.6)	<.001
Shift work	3820 (46.9)	3146 (47.4)	674 (44.8)	.08
Current job shift times, n (%)				<.001
4:00 AM-8:00 AM and 8:00 PM-8:00 PM	1260 (33.0)	1011 (32.1)	249 (37.0)	
8:00 AM-12:00 PM and 8:00 PM-12:00 AM	939 (24.6)	827 (26.3)	112 (16.6)	
12:00 AM-4:00 AM and 12:00 PM-4:00 PM	888 (23.3)	718 (22.8)	170 (25.2)	
Others	733 (19.2)	590 (18.8)	143 (21.2)	
Monthly income (yuan), n (%)				
>8000 (CNY ¥1=US $0.149 as of September 9, 2026)	6725 (82.6)	5405 (81.4)	1320 (87.8)	<.001
Sleep duration^c^ (h), n (%)				<.001
>7	2211 (27.3)	1965 (29.7)	246 (16.4)	
6-<7	2534 (31.2)	2096 (31.7)	438 (29.2)	
5-<6	3011 (37.1)	2284 (34.5)	727 (48.5)	
<5	359 (4.4)	271 (4.1)	88 (5.9)	
Hypertension, n (%)	828 (10.2)	476 (7.2)	352 (23.4)	<.001
Dyslipidemia, n (%)	285 (3.5)	133 (2.0)	152 (10.1)	<.001
DM/IGT^d^, n (%)	222 (2.7)	114 (1.7)	108 (7.2)	<.001

a NAFLD: nonalcoholic fatty liver disease.

b Data are missing for 26 participants.

c Data are missing for 28 participants.

d DM/IGT: diabetes mellitus/impaired glucose tolerance.

### The Prevalence and Associated Factors of NAFLD Among Seafarers

#### Overall Prevalence and Multivariate Regression Analysis

The overall prevalence of NAFLD is 18.5% (1503/8143). Table 2 presents the prevalence and associated factors of NAFLD among seafarers. Overall, the prevalence of NAFLD was significantly higher among seafarers with a BMI 24.0 kg/m² or above (25.9%, 1261/4868 vs 7.2%, 236/3275), chronic noise exposure (19.4%, 1293/6670 vs 13.8%, 204/1473), higher perceived noise impact (slightly: 16.9%, 589/3490; moderate: 19.1%, 359/1879; significant: 22.5%, 511/2273 vs no effect: 7.6%, 38/501), hypertension (42.5%, 352/828 vs 15.7%, 1151/7315), dyslipidemia (53.3%, 152/285 vs 17.2%, 1351/7858), and DM/IGT (48.7%, 108/222 vs. 17.6%, 1395/7921; all *P*<.001). Additionally, participants with NAFLD were more likely to be older, urban residents, highly educated, former smokers, and those with higher income and shorter sleep duration (Table 2).

**Table 2.** Prevalence and associated factors of nonalcoholic fatty liver disease (NAFLD) among seafarers^a^.

Variables	Prevalence	Age-adjusted model^b^		Full-adjusted model^b^	
		aOR^c^ (95% CI)	*P* values^d^	aOR^c^ (95% CI)	*P* values^d^
Overweight, n/N (%)					
No	236/3275 (7.2)	Ref.^e^		Ref.	
Yes	1261/4868 (25.9)	4.24 (3.65‐4.92)	<.001	4.12 (3.53‐4.80)	<.001
Chronic noise exposure, n/N (%)					
No	204/1473 (13.8)	Ref.		Ref.	
Yes	1293/6670 (19.4)	1.39 (1.18‐1.63)	<.001	1.22 (1.03‐1.46)	.02
Perceived noise impact, n/N (%)					
No effect	38/501 (7.6)	Ref.		Ref.	
Slightly	589/3490 (16.9)	2.34 (1.65‐3.31)	<.001	2.15 (1.49‐3.12)	<.001
Moderate	359/1879 (19.1)	2.67 (1.87‐3.81)	<.001	2.31 (1.58‐3.38)	<.001
Significant	511/2273 (22.5)	3.21 (2.26‐4.56)	<.001	2.20 (1.51‐3.21)	<.001
Age groups (y), n/N (%)					
<30	148/1532 (9.7)	Ref.		Ref.	
30-<40	492/2891 (17.0)	1.92 (1.58‐2.33)	<.001	1.22 (0.92‐1.63)	.11
40-<50	413/1881 (22.0)	2.63 (2.15‐3.22)	<.001	1.32 (1.00‐2.33)	.052
≥50	439/1813 (24.2)	2.99 (2.44‐3.65)	<.001	1.25 (1.39‐3.46)	.001
Ethnicity, n/N (%)					
Minor	40/281 (14.2)	Ref.			
Han	1457/7862 (18.5)	1.20 (0.85‐1.69)	.30	—^f^	
Hukou, n/N (%)					
Rural	572/4254 (13.5)	Ref.		Ref.	
Urban	925/3889 (23.8)	1.70 (1.50‐1.93)	<.001	1.37 (1.20‐1.58)	<.001
Marital status, n/N (%)					
Unmarried	197/1840 (10.7)	Ref.		Ref.	
Married	1247/6086 (20.5)	1.27 (0.99‐1.62)	.07	1.02 (0.78‐1.32)	.90
Others	53/217 (24.4)	1.59 (1.07‐2.36)	.02	1.25 (0.81‐1.93)	.31
Bachelor’s degree or higher, n/N (%)					
No	1154/6474 (17.8)	Ref.		Ref.	
Yes	343/1669 (20.5)	1.31 (1.15‐1.51)	<.001	1.08 (0.93‐1.26)	.32
Smoking status, n/N (%)					
Never	652/3973 (16.4)	Ref.		Ref.	
Previous	531/2489 (21.3)	1.26 (1.10‐1.43)	.001	1.18 (1.02‐1.36)	.02
Current	314/1681 (18.7)	1.13 (0.97‐1.32)	.10	1.16 (0.98‐1.36)	.08
Often drinking, n/N (%)					
No	1443/7921 (18.2)	Ref.			
Yes	54/222 (24.3)	1.24 (0.90‐1.70)	.19	—	
Exercise habits, n/N (%)					
No regular exercise routine	952/4894 (19.5)	Ref.		Ref.	
Frequent exercise on board	73/368 (19.8)	1.03 (0.79‐1.35)	.81	1.15 (0.86‐1.54)	.35
Frequent exercise at home	329/1856 (17.7)	0.75 (0.65‐0.87)	<.001	0.73 (0.63‐0.85)	<.001
Frequent exercise both	143/1025 (13.9)	0.56 (0.46‐0.68)	<.001	0.65 (0.52‐0.80)	<.001
Healthy diet, n/N (%)					
No	596/3078 (19.4)	Ref.		Ref.	
Yes	901/5065 (17.8)	0.82 (0.73‐0.92)	.001	0.83 (0.69‐0.99)	.04
Times of job position changes, n/N (%)			.03		
0	1153/6466 (17.8)	Ref.		Ref.	
1	260/1291 (20.1)	1.32 (1.13‐1.54)	<.001	1.40 (1.12‐1.75)	.003
2	84/386 (21.8)	1.43 (1.11‐1.85)	.005	1.25 (0.88‐1.79)	.22
Shift work, n/N (%)					
No	826/4323 (19.1)	Ref.			
Yes	671/3820 (17.6)	1.05 (0.94‐1.19)	.39	—	
Current job shift times^g^, n/N (%)					
4:00 AM-8:00 AM and 4:00 PM-8:00 PM	248/1260 (19.7)	Ref.		—	
8:00 AM-12:00 PM and 8:00 PM-12:00 AM	112/939 (11.9)	0.63 (0.49‐0.81)	<.001	—	
12:00 AM-4:00 AM and 12:00 PM-4:00 PM	170/888 (19.1)	1.01 (0.81‐1.26)	.90	—	
Others	141/733 (19.2)	0.91 (0.72‐1.15)	.44	—	
Monthly income >8000 yuan (CNY ¥1=US $0.149 as of September 9, 2026), n/N (%)					
No	182/1418 (12.8)	Ref.		Ref.	
Yes	1315/6725 (19.6)	1.42 (1.19‐1.69)	<.001	1.25 (0.94‐1.67)	.13
Sleep duration (h), n/N (%)					
>7	246/2211 (11.1)	Ref.		Ref.	
6-<7	436/2534 (17.2)	1.56 (1.32‐1.85)	<.001	1.39 (1.17‐1.66)	<.001
5-<6	725/3011 (24.1)	2.30 (1.97‐2.70)	<.001	1.73 (1.46‐2.06)	<.001
<5	86/359 (24.0)	2.35 (1.78‐3.11)	<.001	1.72 (1.27‐2.33)	<.001
Hypertension, n/N (%)					
No	1151/7315 (15.7)	Ref.		Ref.	
Yes	352/828 (42.5)	3.40 (2.89‐3.99)	<.001	2.17 (1.82‐2.58)	<.001
Dyslipidemia, n/N (%)					
No	1351/7858 (17.2)	Ref.		Ref.	
Yes	152/285 (53.3)	4.56 (3.57‐5.83)	<.001	3.45 (2.63‐4.52)	<.001
DM/IGT^h^, n/N (%)					
No	1395/7921 (17.6)	Ref.		Ref.	
Yes	108/222 (48.7)	3.62 (2.76‐4.77)	<.001	3.50 (2.58‐4.75)	<.001

a Fully covariate-adjusted including those variables with a *P* value <.05 in the age-adjusted analysis. A *P* value <.05 was considered statistically significant.

b Data are shown as age-adjusted and fully covariate-adjusted OR and their 95% CIs.

c aOR: adjusted odds ratio.

d Fully covariate-adjusted including those variables with a *P* value <.05 in the age-adjusted analysis. A *P* value <.05 was considered statistically significant.

e Ref: reference.

f Not applicable. The corresponding variables were not included in the multivariable regression model.

g This variable only describes shift workers and was therefore excluded from the final multivariate regression model.

h DM/IGT: diabetes mellitus/impaired glucose tolerance.

The increased risk of NAFLD was associated with a BMI of 24.0 kg/m² or above (adjusted odds ratio [aOR] 4.12, 95% CI 3.53‐4.80), chronic noise exposure (aOR 1.22, 95% CI 1.03‐1.46), higher perceived noise impact (reference: no effect; slightly: aOR 2.15, 95% CI 1.49‐3.12; moderate: aOR 2.31, 95% CI 1.58‐3.38; significant: aOR 2.20, 95% CI 1.51‐3.21), age of 50 years or above (reference: age <30 years; aOR 1.25, 95% CI 1.39‐3.46), urban residency (aOR 1.37, 95% CI 1.20‐1.58), former smoker (aOR 1.18, 95% CI 1.02‐1.36), one position change (aOR 1.40, 95% CI 1.12‐1.75), shorter sleep duration (reference: >7 h; 6-7 h: aOR 1.39, 95% CI 1.17‐1.66; 5‐6 h: aOR 1.73, 95% CI 1.46‐2.06;<5 hours: aOR 1.72, 95% CI 1.27‐2.33), hypertension (aOR 2.17, 95% CI 1.82‐2.58), dyslipidemia (aOR 3.45, 95% CI 2.63‐4.52), and DM/IGT (aOR 3.50, 95% CI 2.58‐4.75). Conversely, frequent exercise (reference: no regular exercise routine; exercise at home: aOR 0.73, 95% CI 0.63‐0.85; exercise at home and on board: aOR 0.65, 95% CI 0.52‐0.80) and a healthy diet (aOR 0.83, 95% CI 0.69‐0.99) were associated with lower risk of NAFLD.

#### Associations of Noise Exposure and Perceived Noise Impact With NAFLD Across BMI Categories

Across all noise exposure and perceived impact levels, seafarers with a BMI of 24.0 kg/m² or above had a significantly higher prevalence of NAFLD (all *P*<.001; Figure 1A and B).

**Figure 1.**
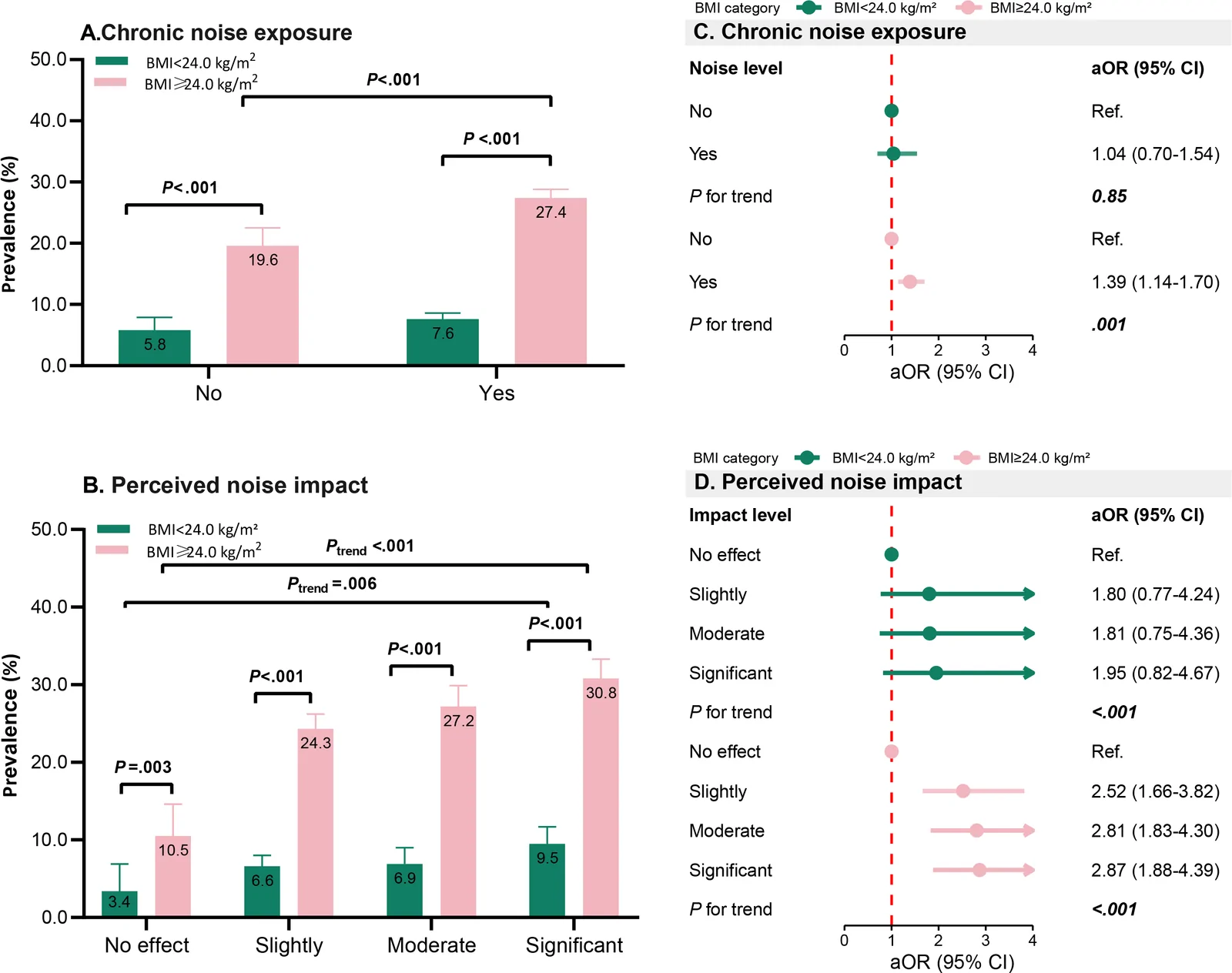
Prevalence and associations of nonalcoholic fatty liver disease (NAFLD) with chronic noise exposure and perceived noise impact stratified by BMI category. (A) and (B) show the prevalence of NAFLD across chronic noise exposure status and perceived noise impact, respectively, stratified by BMI category groups. Bars represent the prevalence (with 95% CIs) of NAFLD. *P* values for trend in (A) and (B) were calculated for both BMI categories, but only the statistically significant values were presented. Forest plots (C) and (D) illustrate the associations of chronic noise exposure and perceived noise impact, respectively, with NAFLD by BMI category. Different colors represent different BMI groups. All multivariate regression models were adjusted for age, household registration, marital status, education, smoking status, physical activity, dietary habits, shift work, monthly income, sleep duration, hypertension, dyslipidemia, diabetes mellitus or impaired glucose tolerance, and BMI (continuous variable). aOR: adjusted odds ratio.

Figure 1A shows the NAFLD prevalence by chronic noise exposure and BMI group. Among the BMI ≥24.0 kg/m² group, those with chronic noise exposure showed a significantly higher prevalence of NAFLD (*P*<.001). However, among the BMI<24.0 kg/m² group, no significant difference in NAFLD prevalence was observed between the noise-exposed and nonexposed groups. Multivariate logistic regression further showed that chronic noise exposure was significantly associated with higher risk of NAFLD in this subgroup (aOR 1.39, 95% CI 1.14‐1.70; Figure 1C). Additionally, NAFLD prevalence rose alongside higher noise exposure in workplace and resting environments, yet no significant association was observed in multivariate regression analyses (Figure S5 in Multimedia Appendix 1).

Figure 1B also shows an uptrend in NAFLD prevalence as perceived noise impact increased in both BMI groups (*P*_trend_=.006 for BMI <24.0 kg/m², and *P*_trend_<.001 for BMI ≥24.0 kg/m²). When perceived noise impact was included as a categorical variable in the model, it was significantly associated with higher risk of NAFLD among the group of BMI ≥24.0 kg/m^2^ (reference: no effect; slightly: aOR 2.52, 95% CI 1.66‐3.82; moderate: aOR 2.81, 95% CI 1.83‐4.30; significant: aOR 2.87 95% CI 1.88‐4.39; Figure 1D). Results stratified by BMI into the normal weight, overweight, and obese subgroups are also presented in Table S3 (Multimedia Appendix 1), which were consistent with the above findings. Significant positive associations with NAFLD were observed only in the overweight and obese subgroups for both chronic noise exposure and perceived noise impact.

#### Combined Associations of Chronic Noise Exposure and Perceived Noise Impact With NAFLD

Figure 2A-C illustrates the trends in NAFLD prevalence across combined subgroups of noise exposure and perceived noise impact in the total population, individuals with a BMI of less than 24.0 kg/m², and individuals with a BMI of 24.0 kg/m² or above, respectively. After full adjustment, among participants with a BMI of less than 24.0 kg/m², none of the subgroups combining noise exposure and elevated perceived noise impact exhibited a significantly elevated NAFLD risk compared with the reference group (no noise exposure and no perceived noise impact; Figure 2E). In contrast, among participants with a BMI of 24.0 kg/m² or above, chronic noise exposure combined with elevated perceived noise impact was significantly associated with higher risk of NAFLD (slightly: aOR 2.72, 95% CI 1.38‐5.34; moderate: aOR 3.02, 95% CI 1.53‐5.98; significant: aOR 2.92, 95% CI 1.49‐5.76). Notably, even in the absence of noise exposure, the highest level of perceived noise impact alone remained significantly associated with increased risk of NAFLD (aOR 2.83, 95% CI 1.27‐6.26; Figure 2F).

**Figure 2.**
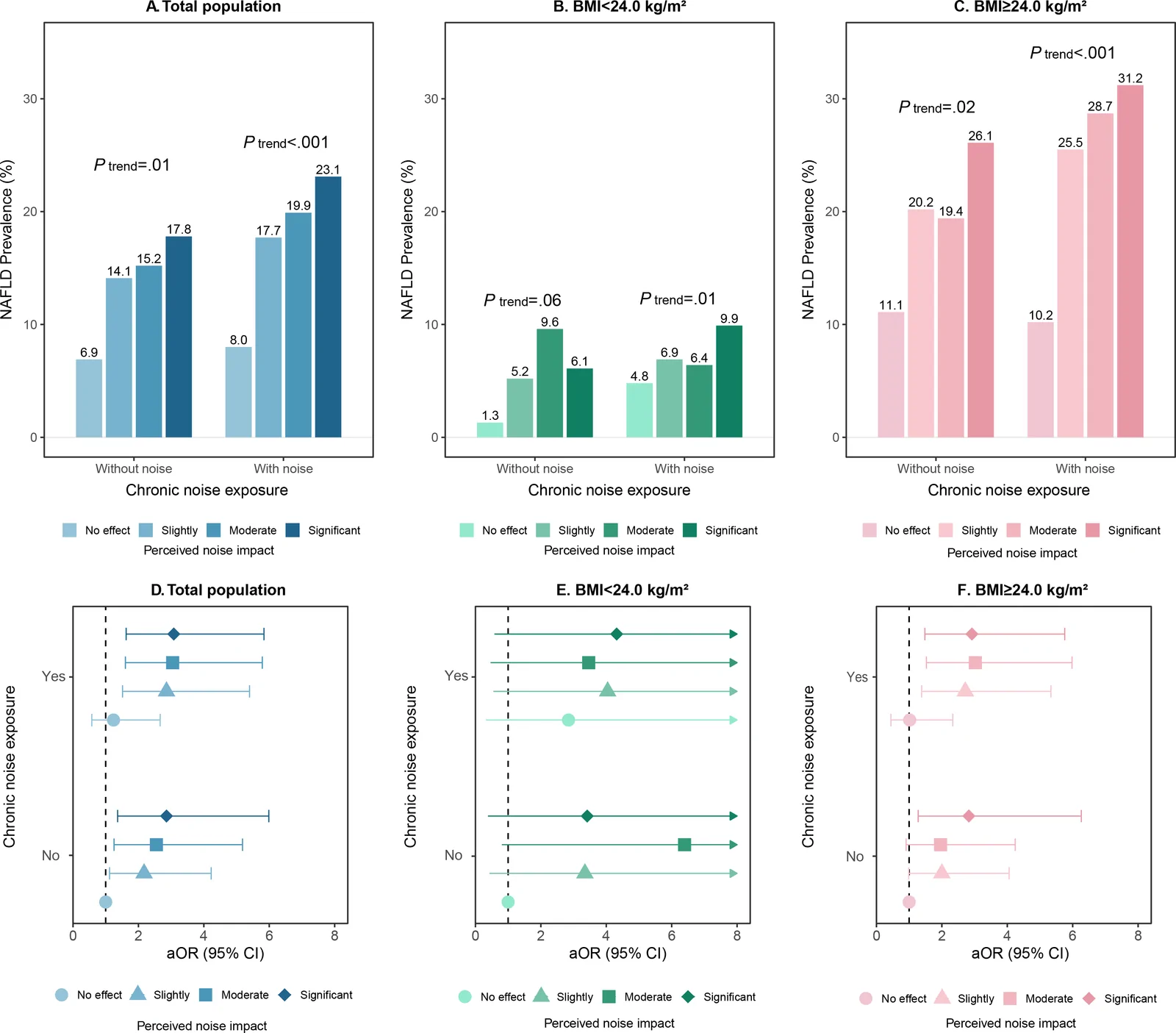
The combined association of chronic noise exposure and perceived noise impact with nonalcoholic fatty liver disease (NAFLD) in total and by BMI category. Panels (A), (B), and (C) show the prevalence of NAFLD stratified by chronic noise exposure and perceived noise impact for all participants and by BMI subgroups, respectively. Forest plots (D), (E), and (F) illustrate the combined association of chronic noise exposure and perceived noise impact with NAFLD for all participants and by BMI category, respectively. All multivariate regression models were adjusted for age, household registration, marital status, education, smoking status, physical activity, dietary habits, shift work, monthly income, sleep duration, hypertension, dyslipidemia, diabetes mellitus or impaired glucose tolerance, and BMI (continuous variable). aOR: adjusted odds ratio.

Figure 3 presents the NAFLD prevalence and the combined associations of workplace or resting-area noise levels and perceived noise impact with NAFLD. Among participants with a BMI of less than 24.0 kg/m², the results remained consistent with our previous observations (Figure 3H,K). However, in the BMI ≥24.0 kg/m² group, elevated perceived noise impact was significantly associated with higher risk of NAFLD, regardless of environmental noise levels in work or rest settings (Figure 3I,L).

**Figure 3.**
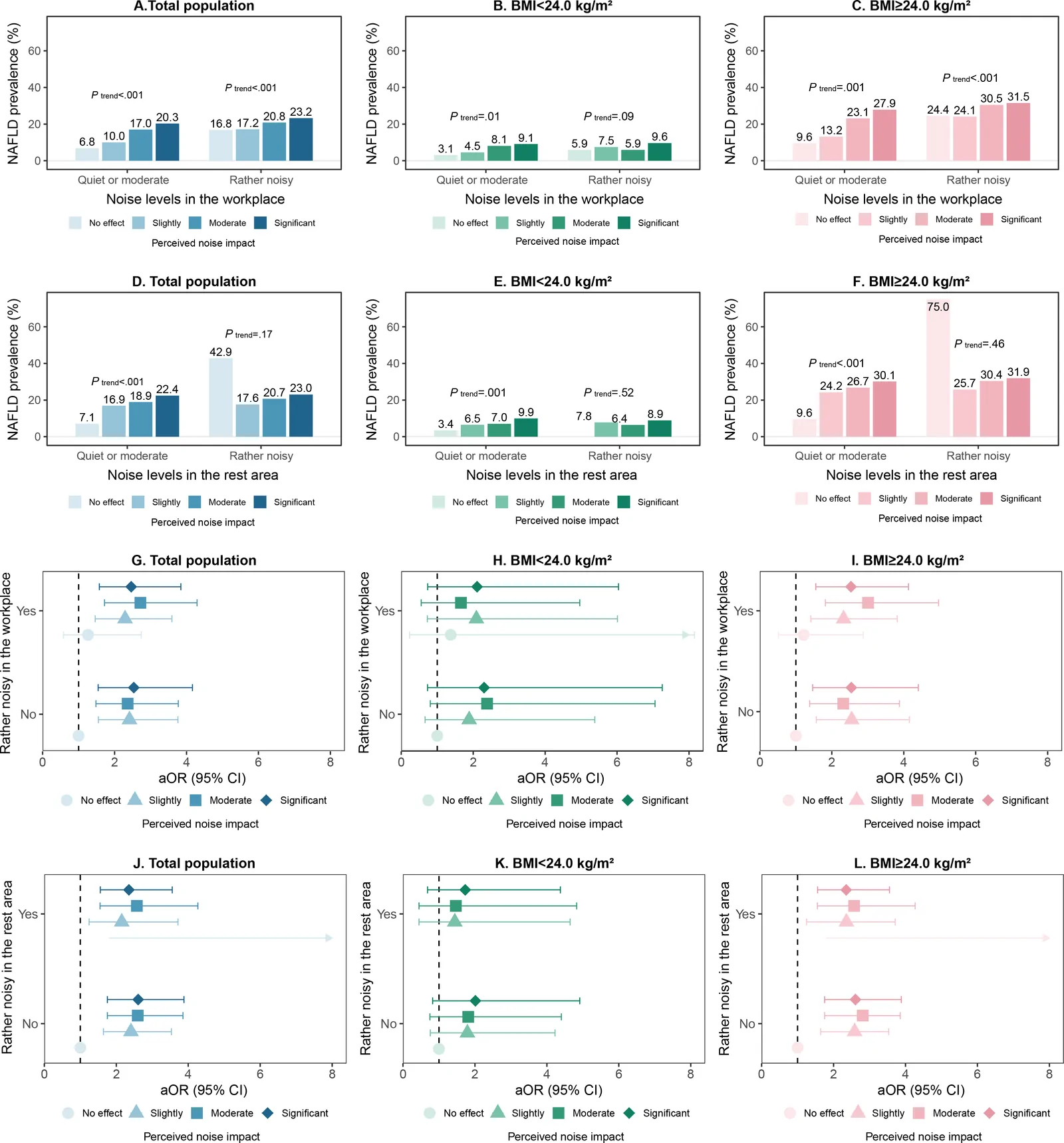
The combined association of noise levels at different places and perceived noise impact with nonalcoholic fatty liver disease (NAFLD) in total and by BMI category. Panels (A), (B), and (C) show the prevalence of NAFLD stratified by noise levels in the workplace and perceived noise impact for all participants and by BMI subgroups, respectively. Panels (D), (E), and (F) show the prevalence of NAFLD stratified by noise levels in the rest area and perceived noise impact for all participants and by BMI subgroups, respectively. Forest plots (G), (H), and (I) illustrate the combined association of noise levels in the workplace and perceived noise impact with NAFLD for all participants and by BMI category, respectively. Forest plots (J), (K), and (L) illustrate the combined association of noise levels in the rest area and perceived noise impact with NAFLD for all participants and by BMI category, respectively. All multivariate regression models were adjusted for age, household registration, marital status, education, smoking status, physical activity, dietary habits, shift work, monthly income, sleep duration, hypertension, dyslipidemia, diabetes mellitus or impaired glucose tolerance, and BMI (continuous variable). aOR: adjusted odds ratio.

#### Interactive Effect of Noise Exposure and Perceived Noise Impact on NAFLD by BMI

Interactive analysis of noise exposure and perceived noise impact could be seen in Table S4 (Multimedia Appendix 1). Among individuals with a BMI of 24.0 kg/m² or above, a significant multiplicative interaction between resting-area noise and perceived noise impact was observed (all *P*<.05). Specifically, the positive association between perceived noise impact and NAFLD was significant only in quiet or moderately noisy resting environments (*P*_trend_<.001), but not under noisy conditions. Moreover, this positive association was consistently observed across all workplace noise levels and chronic noise exposure statuses, regardless of whether perceived noise impact was modeled as a categorical or continuous variable (Table S4 in Multimedia Appendix 1).

### Sensitivity Analysis

To examine the robustness of our findings, we performed sensitivity analysis by excluding participants with self-reported hearing loss. The associations of chronic noise exposure and perceived noise impact with NAFLD across BMI strata (Tables S5-S6 in Multimedia Appendix 1) remained materially unchanged, confirming the stability of our results.

## Discussion

### Key Findings

This is the first large-scale study to estimate the prevalence of NAFLD and its associations with noise exposure, perceived noise impact, and BMI among seafarers. It not only fills a critical gap in maritime occupational noise-related metabolic health research, but also highlights an urgent need for targeted NAFLD prevention strategies to mitigate liver damage in 2.03 million Chinese seafarers and global seafarers more broadly [21]. The overall prevalence of NAFLD among 8143 Chinese seafarers was 18.5% (n=1503), with higher prevalence observed in those with a BMI of 24.0 kg/m² or above, chronic noise exposure, and higher perceived noise impact (Textbox 1). The combined association of chronic noise exposure and perceived noise impact with NAFLD was only observed among individuals with a BMI of 24.0 kg/m² or above. Furthermore, a significant interaction between resting-area noise levels and perceived noise impact in relation to NAFLD was identified specifically in the overweight and obese subgroup.

Textbox 1. **Key messages.**

- We assessed the association of chronic noise exposure, perceived noise exposure, and metabolic status on nonalcoholic fatty liver disease (NAFLD) in seafarers, a crucial occupational group.
- We found evidence that chronic noise exposure, higher perceived noise impact, and their interaction were significantly associated with NAFLD only among seafarers with a BMI higher than 24.0 kg/m^2^.
- Our findings provide novel insights into the potential mechanisms underlying the roles of chronic noise exposure and psychological factors in the development and progression of NAFLD, particularly among individuals with metabolic disorders.

### Comparison With Previous Studies

The prevalence of NAFLD in this study was significantly lower than that reported in the general Chinese population in 2 previous studies (36.7% and 23.6%) [22,23]. This difference is partly attributable to the predominantly manual labor roles of seafarers, which result in higher levels of habitual physical activity compared to the largely sedentary general population. Additionally, the occupational demands of seafaring impose higher physical fitness requirements, necessitating preemployment medical screening. This selection process likely contributes to a “healthy worker effect,” leading to a higher baseline level of health within this occupational group. As seafaring is a strenuous occupation with rigorous physical fitness criteria, only physically qualified individuals can sustain active sea service, rendering currently employed seafarers generally healthier than the general population. Additionally, NAFLD prevalence increases progressively with age. While our study population was predominantly composed of young and middle-aged seafarers, with individuals aged 55 years or above accounting for merely 6.9% (n=563) of the total sample. This relatively young age distribution may further partially explain the lower NAFLD prevalence observed in the present seafarer population.

Previous studies have confirmed that overweight and obesity act as the core pathological basis of NAFLD through triggering insulin resistance and ectopic fat deposition [24,25]. While our study suggests that the noise exposure may synergistically exacerbate NAFLD pathogenesis alongside obesity-related metabolic disorders. As obesity inherently disrupts insulin sensitivity and promotes ectopic lipid accumulation, concurrent noise exposure might amplify hepatic lipid metabolic disturbances via the HPA axis. This pathway elevates circulating cortisol and stimulates sympathetic nerve activity, jointly accelerating the progression of NAFLD [26].

Several studies have reported associations between occupational noise exposure and lipid metabolic disorders, yet direct evidence linking noise exposure to NAFLD, and its combined effects with obesity, remains limited. Studies based on Chinese public institutions and corporate workers, as well as the general US population, have documented a significant association between noise exposure and elevated BMI [27,28]. A previous study involving 4672 male automotive manufacturing workers reported that individuals with cumulative noise exposure of 95 dB(A) or above had a 39% increased risk of NAFLD [29]. Chronic noise exposure may activate the HPA axis, resulting in elevated glucocorticoid levels [8]. Glucocorticoids in turn promote hepatic lipid accumulation by stimulating adipose tissue lipolysis and hepatic de novo lipogenesis, while simultaneously reducing hepatic fatty acid β-oxidation [30], thereby favoring the development of NAFLD.

Interestingly, we observed that higher perceived noise impact was significantly associated with a higher prevalence of NAFLD. Perceived noise impact can be conceptualized as a phenotypic expression of psychological stress in response to environmental noise. It arises from the combined influence of noise stimuli and personal traits and is positively associated with noise sensitivity [31,32]. The positive relationship between perceived noise impact and NAFLD might also be mediated by the chronic HPA axis stress pathway. Long-term perceived noise disturbance may elicit negative emotions and psychological annoyance, thereby activating the HPA axis and promoting excessive cortisol secretion. Previous acoustic research has shown that individuals with high perceived noise exposure report significantly higher levels of negative emotions and remain in a sustained stress state [31], which provides theoretical support for the mechanistic hypothesis of this study. However, given the cross-sectional design of this study, the observed association between perceived noise impact and NAFLD cannot rule out reverse causality; that is, individuals with preexisting NAFLD may be more vulnerable to perceived noise disturbance. To date, some studies have reported a positive association between NAFLD and anxiety, and further targeted research on this topic is warranted.

This study is also the first to investigate the combined associations of chronic noise exposure and perceived noise impact with NAFLD. We found that NAFLD risk was significantly elevated among individuals who reported higher perceived noise impact in relatively quiet environments, or who had both chronic noise exposure and perceived noise impact. This suggests that perceived noise impact may operate independently of noise exposure. Accordingly, environmental noise reduction alone is insufficient to protect this population; psychological stress alleviation and acoustic environment optimization are also needed. Notably, perceived noise impact was significantly associated with higher risk of NAFLD regardless of whether the workplace or living quarters were reported as noisy, underscoring its importance as a psychological stress indicator. In resting areas, even when perceived noise impact was rated as “no effect,” NAFLD risk remained significantly elevated, possibly because metabolic regulation is more vulnerable during rest periods, and residential noise may still disturb metabolic homeostasis with little subjective awareness. Furthermore, a significant multiplicative interaction between noise exposure and perceived noise impact in resting areas suggests that noise-related psychological stress is a key driver of NAFLD.

These associations were observed exclusively among overweight or obese individuals, indicating that this population is more vulnerable to noise-induced stress, which may disrupt hepatic lipid metabolism through chronic stress pathways. Notably, clinical research further supports this biological rationale: body fat distribution and cortisol clearance jointly modulate the association between NAFLD and insulin sensitivity, and higher cortisol clearance is independently associated with reduced insulin sensitivity regardless of BMI [33].

Given that noise-induced psychological stress contributes to metabolic dysfunction, lifestyle interventions that relieve stress and improve metabolism may mitigate NAFLD risk. Currently, physical activities, such as yoga and mindfulness meditation, not only exert positive effects on mood regulation but also contribute to the improvement of inflammation and metabolic status [34,35]. Consistent with this, our study found that regular physical activity and a healthy diet were both significantly associated with a lower prevalence of NAFLD. All the above findings confirm that a healthy lifestyle may effectively improve health simultaneously through multiple pathways.

### Practical Implications for Maritime Occupational Health

This study highlighted the importance of corresponding public health interventions for seafarers. The onboard acoustic environment can be improved by renovating living quarters, reducing mechanical equipment noise, and developing and deploying advanced noise-protection equipment for seafarers. Beyond physical noise control, targeted support for crew members is also critical, such as reasonable shift planning to secure adequate rest and avoid cumulative noise-triggered psychological stress, psychological counseling services, and regular recreational activities. Shipping companies should also establish integrated occupational health management systems featuring routine health education, specialized physical examinations, and personalized intervention programs to sustain seafarers’ physical and mental health.

### Limitations

Several limitations should be considered when interpreting the findings of this study. First, noise exposure was assessed via self-reported measures rather than objective personal dosimetry. While self-reported noise exposure assessment is a practical approach for large-scale epidemiological surveys and has been shown to correlate reasonably well with objective instrumental measurements in previous validation studies, it does not allow for precise quantification of the actual decibel levels experienced by individual seafarers. Second, detailed data on the use of hearing protection devices (HPDs) were not collected. The use of HPDs could attenuate the actual noise exposure intensity and thus may influence the observed effect estimates. Nevertheless, self-perceived noise exposure—even with HPD use—may still reflect the actual exposure level in the current occupational setting and may capture the association with the outcome of interest [36]. However, the absence of both objective dosimetry and HPD usage data in the present study limits our ability to directly compare our findings with quantitative exposure-response relationships reported in the literature. Future investigations incorporating objective noise dosimetry and detailed HPD usage records are needed to more precisely describe the association between occupational noise exposure and NAFLD among seafarers. Third, as seafarers self-reported their fatty liver findings from routine check-ups, which may introduce information bias. Participants might underreport fatty liver due to poor memory or stigma, or alternatively over-attribute liver abnormalities to occupational stress. Although multivariate adjustment for highly comorbid metabolic disorders was performed, it cannot correct for recall errors directly. However, the questionnaire did not explicitly implicate a causal link between noise exposure and liver disease, reducing the likelihood of systematic overreporting driven by participant expectations. We additionally conducted a sensitivity analysis excluding participants with self-reported hearing loss—a subgroup potentially more attentive to noise-related health effects—and found that the ORs, effect directions, and statistical significance of the noise-NAFLD associations were consistent with the primary analysis, suggesting that reporting bias did not materially alter our main findings. Nevertheless, in the absence of objective clinical gold-standard verification or individual-level Kappa concordance tests against medical records, residual recall bias cannot be entirely ruled out. Fourth, the nature of the cross-sectional study design limits its capacity to establish causality. In addition, the sample only comprises Chinese male seafarers. Female participants only accounted for only 0.85% (n=70) of the original sample, consistent with the 1% share of female maritime workers reported in the 2024 survey by the International Maritime Organization and the Women’s International Shipping & Trading Association [37]. Therefore, the results cannot be generalized to female seafarers. Finally, the generalizability of our findings to other populations or occupational settings should be interpreted with caution, as offshore working conditions and living environments differ substantially from land-based jobs.

### Conclusions

In conclusion, this study suggests that chronic noise exposure and perceived noise impact are significantly associated with elevated NAFLD risk among seafarers, particularly individuals with a BMI higher than 24.0 kg/m^2^. The combined associations of chronic noise exposure and perceived noise impact highlight the potential role of environmental and psychological factors in NAFLD. Longitudinal studies are warranted to confirm these associations.

## Supplementary material

10.2196/57844 Multimedia Appendix 1 Supplementary methods, figures, and tables related to the study.

## Abbreviations

aOR: adjusted odds ratio
CSA: China Shipowners’ Association
DM/IGT: diabetes mellitus/impaired glucose tolerance
HPA: hypothalamic-pituitary-adrenal
HPD: hearing protection device
NAFLD: nonalcoholic fatty liver disease
