# Supplementary material for: Association of Noise Exposure, Perceived Noise Impact, and BMI on Nonalcoholic Fatty Liver Disease Among Chinese Seafarers: Nationwide Population-Based Survey
Source: JMIR Public Health Surveill. 2026 Oct 13;12:e57844. doi: 10.2196/57844 (PMC13660749; doi:10.2196/57844)
Supplement: Supplementary file 1 [file publichealth-v12-e57844-s001.docx]

**Supplementary**

**Figure S1** Flowchart **-----------------------------------------------------------------------------------2**

**Figure S2** Distribution of different kinds of noise exposure and noise impact among all seafarers**---------------------------------------------------------------------------------------------------3**

**Figure S3** Distribution of different kinds of noise exposure and noise impact among seafarers with BMI<24.0 kg/m^2^**-------------------------------------------------------------------------------------4**

**Figure S4** Distribution of different kinds of noise exposure and noise impact among seafarers with BMI≥24.0 kg/m^2^**-----------------------------------------------------------------------------------5**

**Figure S5** Prevalence and associations of NAFLD with noise levels at workplace and rest area stratified by BMI category -**------------------------------------------------------------------------------6**

**Table S1** Total variance explained for Harman’s single-factor common method bias test **--------7**

**Table S2** Comparison of characteristics between included males and excluded females**----10**

**Table S3** Factors associated with NAFLD among Chinese seafarers by three BMI category **------------------------------------------------------------------------------------------------------------12**

**Table S4** Interactive effect of noise exposure and perceived noise impact with NAFLD among seafarers by BMI category-**-------------------------------------------------------------------13**

**Table S5** Sensitivity analysis for the independent associations of chronic noise exposure and perceived noise impact with NAFLD by excluding participants who reported hearing loss**-15**

**Table S6** Sensitivity analysis for the combined associations of noise exposure and perceived noise impact with NAFLD by excluding participants who reported hearing loss-**------------16**

All subjects recruited from 1^st^ March to 31^st^ May 2024

N=8223

n=29,823

The subjects included

N=8143

80 Subjects excluded

2 Other liver disease

1 Data error

7 BMI data missing

70 Female

### Figure S1. Flowchart

### Figure S2 Distribution of different kinds of noise exposure and noise impact among all seafarers. All *P* values were calculated from Chi-square tests. BMI, Body Mass Index; DM, Diabetes mellitus; IGT, Impaired glucose tolerance. Three distinct colors in the figure represent workplace noise levels, rest-area noise levels, and perceived noise impact, respectively.

### Figure S3 Distribution of different kinds of noise exposure and noise impact among seafarers with BMI<24.0 kg/m^2^. All P values were calculated from Chi-square tests. BMI, Body Mass Index; DM, Diabetes mellitus; IGT, Impaired glucose tolerance. Three distinct colors in the figure represent workplace noise levels, rest-area noise levels, and perceived noise impact, respectively.

### Figure S4 Distribution of different kinds of noise exposure and noise impact among seafarers with BMI≥24.0 kg/m^2^. All *P* values were calculated from Chi-square tests. BMI, Body Mass Index; DM, Diabetes mellitus; IGT, Impaired glucose tolerance. Three distinct colors in the figure represent workplace noise levels, rest-area noise levels, and perceived noise impact, respectively.

**Figure S5 Prevalence and associations of NAFLD with noise levels at workplace and rest area stratified by BMI category**. (A) and (B) showed the prevalence of NAFLD across noise levels at workplace and rest area stratified by BMI category groups, respectively. All multivariate regression models were adjusted for age, household registration, marital status, education, smoking status, physical activity, dietary habits, shift work, monthly income, sleep duration, hypertension, dyslipidemia, diabetes mellitus or impaired glucose tolerance and BMI (continuous variable). BMI, Body Mass Index; OR, adjusted Odds Ratio; NAFLD, Non-Alcoholic Fatty Liver Disease.

### Table S1 Total variance explained for Harman’s single-factor common method bias test

| **Factor** | **Eigenvalue** | **Difference** | **Proportion** | **Cumulative** |
| --- | --- | --- | --- | --- |
| Factor1 | 24.5214 | 18.33843 | 0.1994 | 0.1994 |
| Factor2 | 6.18297 | 2.20062 | 0.0503 | 0.2496 |
| Factor3 | 3.98235 | 0.68485 | 0.0324 | 0.282 |
| Factor4 | 3.2975 | 0.59277 | 0.0268 | 0.3088 |
| Factor5 | 2.70474 | 0.34848 | 0.022 | 0.3308 |
| Factor6 | 2.35625 | 0.19168 | 0.0192 | 0.35 |
| Factor7 | 2.16458 | 0.10447 | 0.0176 | 0.3676 |
| Factor8 | 2.06011 | 0.058 | 0.0167 | 0.3843 |
| Factor9 | 2.00211 | 0.10994 | 0.0163 | 0.4006 |
| Factor10 | 1.89217 | 0.0881 | 0.0154 | 0.416 |
| Factor11 | 1.80407 | 0.11632 | 0.0147 | 0.4306 |
| Factor12 | 1.68776 | 0.15344 | 0.0137 | 0.4444 |
| Factor13 | 1.53432 | 0.02014 | 0.0125 | 0.4568 |
| Factor14 | 1.51418 | 0.04319 | 0.0123 | 0.4691 |
| Factor15 | 1.47098 | 0.0632 | 0.012 | 0.4811 |
| Factor16 | 1.40778 | 0.06495 | 0.0114 | 0.4925 |
| Factor17 | 1.34284 | 0.02411 | 0.0109 | 0.5035 |
| Factor18 | 1.31872 | 0.07654 | 0.0107 | 0.5142 |
| Factor19 | 1.24218 | 0.01106 | 0.0101 | 0.5243 |
| Factor20 | 1.23112 | 0.02501 | 0.01 | 0.5343 |
| Factor21 | 1.20611 | 0.03354 | 0.0098 | 0.5441 |
| Factor22 | 1.17257 | 0.03581 | 0.0095 | 0.5536 |
| Factor23 | 1.13675 | 0.0449 | 0.0092 | 0.5629 |
| Factor24 | 1.09185 | 0.02523 | 0.0089 | 0.5718 |
| Factor25 | 1.06662 | 0.00563 | 0.0087 | 0.5804 |
| Factor26 | 1.06099 | 0.02545 | 0.0086 | 0.589 |
| Factor27 | 1.03554 | 0.02578 | 0.0084 | 0.5975 |
| Factor28 | 1.00977 | 0.0123 | 0.0082 | 0.6057 |
| Factor29 | 0.99746 | 0.01976 | 0.0081 | 0.6138 |
| Factor30 | 0.9777 | 0.0121 | 0.0079 | 0.6217 |
| Factor31 | 0.9656 | 0.00889 | 0.0079 | 0.6296 |
| Factor32 | 0.95672 | 0.00704 | 0.0078 | 0.6374 |
| Factor33 | 0.94967 | 0.01535 | 0.0077 | 0.6451 |
| Factor34 | 0.93432 | 0.02532 | 0.0076 | 0.6527 |
| Factor35 | 0.90901 | 0.0157 | 0.0074 | 0.6601 |
| Factor36 | 0.8933 | 0.01429 | 0.0073 | 0.6673 |
| Factor37 | 0.87902 | 0.01344 | 0.0071 | 0.6745 |
| Factor38 | 0.86558 | 0.00677 | 0.007 | 0.6815 |
| Factor39 | 0.85881 | 0.01701 | 0.007 | 0.6885 |
| Factor40 | 0.8418 | 0.01905 | 0.0068 | 0.6953 |
| Factor41 | 0.82275 | 0.00992 | 0.0067 | 0.702 |
| Factor42 | 0.81283 | 0.00844 | 0.0066 | 0.7086 |
| Factor43 | 0.80439 | 0.00975 | 0.0065 | 0.7152 |
| Factor44 | 0.79464 | 0.00682 | 0.0065 | 0.7216 |
| Factor45 | 0.78782 | 0.0062 | 0.0064 | 0.728 |
| Factor46 | 0.78162 | 0.02158 | 0.0064 | 0.7344 |
| Factor47 | 0.76003 | 0.00369 | 0.0062 | 0.7406 |
| Factor48 | 0.75635 | 0.00764 | 0.0061 | 0.7467 |
| Factor49 | 0.74871 | 0.01254 | 0.0061 | 0.7528 |
| Factor50 | 0.73618 | 0.01886 | 0.006 | 0.7588 |
| Factor51 | 0.71732 | 0.00705 | 0.0058 | 0.7646 |
| Factor52 | 0.71027 | 0.01762 | 0.0058 | 0.7704 |
| Factor53 | 0.69265 | 0.00773 | 0.0056 | 0.776 |
| Factor54 | 0.68493 | 0.00708 | 0.0056 | 0.7816 |
| Factor55 | 0.67785 | 0.00633 | 0.0055 | 0.7871 |
| Factor56 | 0.67152 | 0.0049 | 0.0055 | 0.7926 |
| Factor57 | 0.66662 | 0.01725 | 0.0054 | 0.798 |
| Factor58 | 0.64937 | 0.00871 | 0.0053 | 0.8033 |
| Factor59 | 0.64066 | 0.01117 | 0.0052 | 0.8085 |
| Factor60 | 0.62949 | 0.00741 | 0.0051 | 0.8136 |
| Factor61 | 0.62208 | 0.01574 | 0.0051 | 0.8187 |
| Factor62 | 0.60633 | 0.00407 | 0.0049 | 0.8236 |
| Factor63 | 0.60226 | 0.01633 | 0.0049 | 0.8285 |
| Factor64 | 0.58594 | 0.00443 | 0.0048 | 0.8333 |
| Factor65 | 0.58151 | 0.01534 | 0.0047 | 0.838 |
| Factor66 | 0.56617 | 0.00601 | 0.0046 | 0.8426 |
| Factor67 | 0.56016 | 0.00744 | 0.0046 | 0.8471 |
| Factor68 | 0.55272 | 0.00737 | 0.0045 | 0.8516 |
| Factor69 | 0.54535 | 0.00744 | 0.0044 | 0.8561 |
| Factor70 | 0.53791 | 0.01036 | 0.0044 | 0.8604 |
| Factor71 | 0.52755 | 0.00528 | 0.0043 | 0.8647 |
| Factor72 | 0.52228 | 0.01783 | 0.0042 | 0.869 |
| Factor73 | 0.50445 | 0.00759 | 0.0041 | 0.8731 |
| Factor74 | 0.49686 | 0.0101 | 0.004 | 0.8771 |
| Factor75 | 0.48676 | 0.00513 | 0.004 | 0.8811 |
| Factor76 | 0.48164 | 0.01031 | 0.0039 | 0.885 |
| Factor77 | 0.47133 | 0.00274 | 0.0038 | 0.8888 |
| Factor78 | 0.46858 | 0.00765 | 0.0038 | 0.8926 |
| Factor79 | 0.46093 | 0.00359 | 0.0037 | 0.8964 |
| Factor80 | 0.45734 | 0.02051 | 0.0037 | 0.9001 |
| Factor81 | 0.43683 | 0.00695 | 0.0036 | 0.9036 |
| Factor82 | 0.42988 | 0.00428 | 0.0035 | 0.9071 |
| Factor83 | 0.4256 | 0.00104 | 0.0035 | 0.9106 |
| Factor84 | 0.42456 | 0.00912 | 0.0035 | 0.9141 |
| Factor85 | 0.41544 | 0.01095 | 0.0034 | 0.9174 |
| Factor86 | 0.40449 | 0.01315 | 0.0033 | 0.9207 |
| Factor87 | 0.39134 | 0.01371 | 0.0032 | 0.9239 |
| Factor88 | 0.37764 | 0.00818 | 0.0031 | 0.927 |
| Factor89 | 0.36945 | 0.00293 | 0.003 | 0.93 |
| Factor90 | 0.36652 | 0.0028 | 0.003 | 0.933 |
| Factor91 | 0.36372 | 0.01004 | 0.003 | 0.9359 |
| Factor92 | 0.35368 | 0.00618 | 0.0029 | 0.9388 |
| Factor93 | 0.3475 | 0.00501 | 0.0028 | 0.9416 |
| Factor94 | 0.34249 | 0.01141 | 0.0028 | 0.9444 |
| Factor95 | 0.33108 | 0.00724 | 0.0027 | 0.9471 |
| Factor96 | 0.32384 | 0.00455 | 0.0026 | 0.9497 |
| Factor97 | 0.31929 | 0.00665 | 0.0026 | 0.9523 |
| Factor98 | 0.31263 | 0.0046 | 0.0025 | 0.9549 |
| Factor99 | 0.30804 | 0.00723 | 0.0025 | 0.9574 |
| Factor100 | 0.30081 | 0.00193 | 0.0024 | 0.9598 |
| Factor101 | 0.29888 | 0.00537 | 0.0024 | 0.9622 |
| Factor102 | 0.29352 | 0.01214 | 0.0024 | 0.9646 |
| Factor103 | 0.28138 | 0.00884 | 0.0023 | 0.9669 |
| Factor104 | 0.27254 | 0.00438 | 0.0022 | 0.9691 |
| Factor105 | 0.26817 | 0.00276 | 0.0022 | 0.9713 |
| Factor106 | 0.26541 | 0.00185 | 0.0022 | 0.9735 |
| Factor107 | 0.26355 | 0.00887 | 0.0021 | 0.9756 |
| Factor108 | 0.25469 | 0.00227 | 0.0021 | 0.9777 |
| Factor109 | 0.25241 | 0.00573 | 0.0021 | 0.9797 |
| Factor110 | 0.24668 | 0.00767 | 0.002 | 0.9817 |
| Factor111 | 0.23901 | 0.01227 | 0.0019 | 0.9837 |
| Factor112 | 0.22674 | 0.004 | 0.0018 | 0.9855 |
| Factor113 | 0.22274 | 0.00348 | 0.0018 | 0.9873 |
| Factor114 | 0.21926 | 0.00148 | 0.0018 | 0.9891 |
| Factor115 | 0.21778 | 0.0111 | 0.0018 | 0.9909 |
| Factor116 | 0.20668 | 0.01457 | 0.0017 | 0.9926 |
| Factor117 | 0.19211 | 0.00352 | 0.0016 | 0.9941 |
| Factor118 | 0.1886 | 0.00569 | 0.0015 | 0.9957 |
| Factor119 | 0.18291 | 0.00944 | 0.0015 | 0.9971 |
| Factor120 | 0.17347 | 0.01632 | 0.0014 | 0.9986 |
| Factor121 | 0.15715 | 0.14672 | 0.0013 | 0.9998 |
| Factor122 | 0.01043 | 0.00082 | 0.0001 | 0.9999 |
| Factor123 | 0.00961 | . | 0.0001 | 1.0000 |

### Table S2 Comparison of characteristics between included males and excluded females

| Variables | Total  (*n*=8213) | Males  (*n*=8143) | Females  (*n*=70) | *P* value |
| --- | --- | --- | --- | --- |
| BMI≥24.0 kg/m^2^ | 4886(59.5) | 4868(59.8) | 18(25.7) | <0.001 |
| Noise exposure during work | 6710(81.7) | 6670(81.9) | 40(57.1) | <0.001 |
| Impact of noise on the rest and sleep |  |  |  | 0.148 |
| No effect | 509(6.2) | 501(6.2) | 8(11.4) |  |
| Slightly | 3524(42.9) | 3490(42.9) | 34(48.6) |  |
| Moderate | 1891(23.0) | 1879(23.1) | 12(17.1) |  |
| Significant | 2289(27.9) | 2273(27.9) | 16(22.9) |  |
| Age groups^✝^ |  |  |  | <0.001 |
| <30 | 1564(19.1) | 1532(18.9) | 32(45.7) |  |
| 30~<40 | 2908(35.5) | 2891(35.6) | 17(24.3) |  |
| 40~<50 | 1895(23.2) | 1883(23.2) | 14(20.0) |  |
| ≥50 | 1820(22.2) | 1813(22.3) | 7(10.0) |  |
| Ethnicity |  |  |  | 0.02 |
| Minor | 287(3.5) | 281(3.5) | 6(8.6) |  |
| Han | 7926(96.5) | 7862(96.5) | 64(91.4) |  |
| Hukou |  |  |  | 0.185 |
| Rural | 4285(52.2) | 4254(52.2) | 31(44.3) |  |
| Urban | 3928(47.8) | 3889(47.8) | 39(55.7) |  |
| Marital status |  |  |  | <0.001 |
| Unmarried | 1872(22.8) | 1840(22.6) | 32(45.7) |  |
| Married | 6117(74.5) | 6086(74.7) | 31(44.3) |  |
| Others | 224(2.7) | 217(2.7) | 7(10.0) |  |
| Bachelor's degree or higher | 1678(20.4) | 1669(20.5) | 9(12.9) | 0.114 |
| Smoking status |  |  |  | <0.001 |
| Never | 4026(49.0) | 3973(48.8) | 53(75.7) |  |
| Previous | 2496(30.4) | 2489(30.6) | 7(10.0) |  |
| Current | 1691(20.6) | 1681(20.6) | 10(14.3) |  |
| Often drinking | 223(2.7) | 222(2.7) | 1(1.4) | 0.506 |
| Daily alcohol intake | 0(0-0.68) | 0(0-0.68) | 0(0-0) | 0.721 |
| Exercise habits |  |  |  | 0.001 |
| No regular exercise routine | 4952(60.3) | 4894(60.1) | 58(82.9) |  |
| Frequent exercise on board | 372(4.5) | 368(4.5) | 4(5.7) |  |
| Frequent exercise at home | 1862(22.7) | 1856(22.8) | 6(8.6) |  |
| Frequent exercise both | 1027(12.5) | 1025(12.6) | 2(2.9) |  |
| Unhealthy diet | 3125(38.1) | 3078(37.8) | 47(67.1) | <0.001 |
| Times of job position changes |  |  |  | 0.620 |
| 0 | 6521(79.4) | 6466(79.4) | 55(78.6) |  |
| 1 | 1301(15.8) | 1291(15.9) | 10(14.3) |  |
| 2 | 391(4.8) | 386(4.7) | 5(7.1) |  |
| Years on current job, median (IQR) | 4(2-10) | 4(2-10) | 3(1-8) | 0.352 |
| Months per shipboard duty, mean (SD) | 7.6±1.5 | 7.6±1.5 | 7.2±1.2 | 0.416 |
| Daily work hours on a ship, mean (SD) | 8.7±1.4 | 8.7±1.4 | 8.5±1.3 | 0.528 |
| Shift work | 3862(47.0) | 3820(46.9) | 42(60.0) | 0.029 |
| Current job shift times^†^ |  |  |  | <0.001 |
| 04:00 - 08:00 and 16:00 - 20:00 | 1267(32.8) | 1260(33.0) | 7(16.7) |  |
| 08:00 - 12:00 and 20:00 - 24:00 | 948(24.6) | 939(24.6) | 9(21.4) |  |
| 24:00 - 04:00 and 12:00 - 16:00 | 889(23.0) | 888(23.3) | 1(2.4) |  |
| Others | 758(19.6) | 733(19.2) | 25(59.5) |  |
| Monthly income>8000 | 6750(82.2) | 6725(82.6) | 25(35.7) | <0.001 |
| Sleep duration, h^‡^ |  |  |  | 0.11 |
| >7 | 2233(27.3) | 2211(27.3) | 22(31.9) |  |
| 6-<7 | 2548(31.1) | 2534(31.2) | 14(20.3) |  |
| 5-<6 | 3038(37.1) | 3011(37.1) | 27(39.1) |  |
| <5 | 365(4.5) | 359(4.4) | 6(8.7) |  |
| Hypertension | 832(10.1) | 828(10.2) | 4(5.7) | 0.219 |
| Dyslipidemia | 285(3.5) | 285(3.5) | 0(0.0) | 0.111 |
| DM or IGT | 223(2.7) | 222(2.7) | 1(1.4) | 0.506 |

Individuals excluded for the following reasons were not included in the analysis: 1 data error

2 other liver disease, 7 missing BMI data.

^✝^ 26 missing observations among males.

^†^ Shift schedules were only analyzed among shift workers.

^‡^ 28 missing observations among males and 1 missing observation among females.

### Table S3 Factors associated with NAFLD among Chinese seafarers by three BMI category

| Variables | BMI<24.0 kg/m^2^ | |  | | BMI 24.0~27.9 kg/m^2^ | |  | BMI≥28.0 kg/m^2^ | |
| --- | --- | --- | --- | --- | --- | --- | --- | --- | --- |
|  | OR(95%CI) | P value |  | OR(95%CI) | | P value |  | OR(95%CI) | P value |
| Chronic noise exposure |  |  |  |  | |  |  |  |  |
| No | Ref. | - |  | Ref. | | - |  | Ref. | - |
| Yes | 1.04(0.70-1.54) | 0.853 |  | 1.28(1.01-1.63) | | **0.04** |  | 1.68(1.18-2.39) | **0.004** |
| Noise level in the rest area |  |  |  |  | |  |  |  |  |
| Quiet | Ref. | - |  | Ref. | | - |  | Ref. | - |
| Moderate | 0.85(0.32-2.23) | 0.74 |  | 1.29(0.67-2.47) | | 0.445 |  | 1.27(0.41-3.91) | 0.681 |
| Rather noisy | 0.85(0.33-2.22) | 0.738 |  | 1.41(0.74-2.69) | | 0.301 |  | 1.49(0.48-4.59) | 0.487 |
| *P* for trend | 0.86(0.66-1.12) | 0.258 |  | 1.03(0.88-1.20) | | 0.728 |  | 1.25(0.97-1.61) | 0.086 |
| Noise level in the workplace |  |  |  |  | |  |  |  |  |
| Quiet | Ref. | - |  | Ref. | | - |  | Ref. | - |
| Moderate | 0.79(0.52-1.20) | 0.271 |  | 1.10(0.85-1.44) | | 0.464 |  | 1.18(0.80-1.74) | 0.396 |
| Rather noisy | 0.73(0.43-1.22) | 0.227 |  | 1.08(0.78-1.48) | | 0.644 |  | 1.56(0.94-2.58) | 0.085 |
| *P* for trend | 0.98(0.75-1.28) | 0.886 |  | 1.11(0.95-1.30) | | 0.198 |  | 1.18(0.92-1.52) | 0.190 |
| Perceived noise impact |  |  |  |  | |  |  |  |  |
| No effect | Ref. | - |  | Ref. | | - |  | Ref. | - |
| Slightly | 1.8(0.77-4.24) | 0.176 |  | 2.57(1.49-4.42) | | **0.001** |  | 2.36(1.23-4.53) | **0.009** |
| Moderate | 1.81(0.75-4.36) | 0.186 |  | 2.61(1.50-4.55) | | **0.001** |  | 3.3(1.69-6.47) | **<0.001** |
| Significant | 1.95(0.82-4.67) | 0.132 |  | 2.74(1.58-4.75) | | **<0.001** |  | 3.2(1.62-6.29) | **0.001** |
| *P* for trend | 1.08(0.93-1.26) | 0.312 |  | 1.10(1.01-1.21) | | **0.036** |  | 1.28(1.10-1.48) | **0.001** |

All multivariate regression models were adjusted for age, ethnicity, household registration, marital status, education, smoking, alcohol consumption, physical activity, diet, shift work, income, sleep duration, hypertension, dyslipidemia, diabetes mellitus or impaired glucose tolerance and BMI (continuous variable). BMI, Body Mass Index; OR, adjusted Odds Ratios; NAFLD, Non-Alcoholic Fatty Liver Disease.

**Table S4 Interactive effect of noise exposure and perceived noise impact with NAFLD among seafarers by BMI category**

| **Subgroup** | **Impact of noise on the rest and sleep** | | | | ***P for trend***^✝^ |
| --- | --- | --- | --- | --- | --- |
|  | **No effect**  **OR (95%CI)** | **Slightly**  **OR (95%CI)** | **Moderate**  **OR (95%CI)** | **Significant**  **OR (95%CI)** |  |
| **BMI<24.0 kg/m^2^** |  |  |  |  |  |
| **Chronic noise exposure** |  |  |  |  |  |
| No | Ref. | 3.72(0.44-31.41) | 7.60(0.87-66.58) | 3.13(0.31-31.39) | 0.259 |
| Yes | Ref. | 1.49(0.58-3.85) | 1.28(0.48-3.40) | 1.61(0.62-4.22) | 0.500 |
| ***P for interaction***^☨^ |  | 0.457 | 0.155 | 0.505 |  |
| **Noise level in the working places** |  |  |  |  |  |
| Quiet or moderate | Ref. | 1.68(0.58-4.88) | 2.06(0.68-6.23) | 1.89(0.58-6.19) | 0.293 |
| Rather noisy | Ref. | 1.67(0.37-7.49) | 1.36(0.29-6.23) | 1.75(0.39-7.80) | 0.632 |
| ***P for interaction***^☨^ |  | 0.816 | 0.476 | 0.672 |  |
| **Noise level in the resting places** |  |  |  |  |  |
| Quiet or moderate | Ref. | 1.76(0.74-4.16) | 1.80(0.74-4.38) | 1.99(0.81-4.87) | 0.262 |
| Rather noisy |  | - | 0.95(0.36-2.51) | 0.89(0.34-2.36) | 0.786 |
| ***P for interaction***^☨^ |  |  | 0.896 | 0.909 |  |
| **BMI≥24.0 kg/m^2^** |  |  |  |  |  |
| **Chronic noise exposure** |  |  |  |  |  |
| No | Ref. | 2.02(0.97-4.21) | 2.01(0.90-4.48) | 2.89(1.26-6.66)* | **0.035** |
| Yes | Ref. | 2.73(1.62-4.59)*** | 3.05(1.80-5.18)*** | 2.94(1.74-4.98)*** | **0.015** |
| ***P for interaction***^☨^ |  | 0.516 | 0.381 | 0.973 |  |
| **Noise level in the working places** |  |  |  |  |  |
| Quiet or moderate | Ref. | 2.63(1.58-4.35)*** | 2.41(1.41-4.11)** | 2.98(1.68-5.30)*** | **0.021** |
| Rather noisy | Ref. | 2.40(1.10-5.22)* | 3.11(1.42-6.80)** | 2.84(1.31-6.16)** | **0.028** |
| ***P for interaction***^☨^ |  | 0.741 | 0.7171 | 0.756 |  |
| **Noise level in the resting places** |  |  |  |  |  |
| Quiet or moderate | Ref. | 2.73(1.76-4.22)*** | 3.00(1.92-4.70)*** | 3.15(2.00-4.95)*** | **<0.001** |
| Rather noisy | Ref. | 0.15(0.01-1.79) | 0.18(0.02-2.11) | 0.19(0.02-2.16) | 0.726 |
| ***P for interaction***^☨^ |  | **0.021** | **0.025** | **0.020** |  |

Note: * indicated *P*<0.05; ** indicated *P*<0.01; ** indicated *P*<0.001. BMI, Body Mass Index; OR, adjusted Odds Ratios; NAFLD, Non-Alcoholic Fatty Liver Disease.

^✝^The *P for trend* is calculated as the p-value corresponding to the odds ratio when the variable noise impact is included into the model as a continuous variable.

^☨^The *P for interaction* indicated the statistical significance of the interaction term between noise exposure levels (categorical) and perceived noise impact levels (categorical) in the regression model.

All multivariate regression models were adjusted for age, household registration, marital status, education, smoking status, physical activity, dietary habits, shift work, monthly income, sleep duration, hypertension, dyslipidemia, diabetes mellitus or impaired glucose tolerance and BMI (continuous variable). Multiplicative interaction terms between noise exposure and perceived noise impact were tested by including product terms in the regression models. The interaction *P*-values presented in the table correspond to these multiplicative interaction terms.

**Table S5 Sensitivity analysis for the independent associations of chronic noise exposure and perceived noise impact with NAFLD by excluding participants who reported hearing loss**

| Variables | Age-adjusted model^✝^ | |  | Full-adjusted model^✝^ | |
| --- | --- | --- | --- | --- | --- |
|  | OR (95%CI) | *P* values |  | OR (95%CI) | *P* values |
| Chronic noise exposure |  |  |  |  |  |
| No | Ref. |  |  | Ref. |  |
| Yes | 1.35(1.15-1.60) | <0.001 |  | 1.22(1.02-1.46) | 0.031 |
| Perceived noise impact | |  |  |  |  |
| No effect | Ref. |  |  | Ref. |  |
| Slightly | 2.22(1.56-3.16) | <0.001 |  | 2.02(1.39-2.94) | <0.001 |
| Moderate | 2.56(1.78-3.68) | <0.001 |  | 2.20(1.50-3.24) | <0.001 |
| Significant | 3.07(2.15-4.40) | <0.001 |  | 2.08(1.42-3.06) | <0.001 |

Note: BMI, Body Mass Index; OR, adjusted Odds Ratios; CI, confidence interval; NAFLD, Non-Alcoholic Fatty Liver Disease.

Full-adjusted regression models were adjusted for age, household registration, marital status, education, smoking status, physical activity, dietary habits, shift work, monthly income, sleep duration, hypertension, dyslipidemia, diabetes mellitus or impaired glucose tolerance and BMI subgroup.

**Table S6 Sensitivity analysis for the combined associations of noise exposure and perceived noise impact with NAFLD by excluding participants who reported hearing loss**

| **Combined variables** | **OR (95%CI)** | | |
| --- | --- | --- | --- |
|  | **Overall** | **BMI<24.0kg/m2** | **BMI≥24.0kg/m2** |
| **Chronic noise exposure-perceived noise impact** | |  |  |
| No exposure-no effect | Reference | Reference | Reference |
| No exposure-slightly | 2.02(1.04-3.93)* | 3.26(0.42-25.37) | 1.82(0.9-3.71) |
| No exposure-moderate | 2.38(1.17-4.86)* | 6.05(0.76-48.14) | 1.82(0.84-3.96) |
| No exposure-significant | 2.68(1.27-5.67)* | 3.46(0.39-30.94) | 2.64(1.17-5.92)* |
| Exposed-no effect | 1.26(0.58-2.72) | 2.57(0.28-23.79) | 1.06(0.46-2.43) |
| Exposed-slightly | 2.64(1.39-4.99)** | 3.8(0.51-28.01) | 2.48(1.26-4.89)** |
| Exposed-moderate | 2.85(1.5-5.44)** | 3.1(0.41-23.35) | 2.84(1.43-5.64)** |
| Exposed-significant | 2.88(1.52-5.47)** | 4.36(0.59-32.43) | 2.67(1.35-5.28)** |
| **Noise in the workplace-perceived noise impact** | |  |  |
| Quiet or moderate-no effect | Reference | Reference | Reference |
| Quiet or moderate-slightly | 2.37(1.50-3.74)*** | 2.29(0.69-7.55) | 2.43(1.48-4.00)*** |
| Quiet or moderate-moderate | 2.43(1.50-3.93)*** | 2.96(0.87-10.14) | 2.33(1.38-3.96)** |
| Quiet or moderate-significant | 3.01(1.8-5.04)*** | 3.05(0.85-11.00) | 3.11(1.76-5.47)*** |
| Rather noisy-no effect | 1.45(0.65-3.23) | 2.41(0.37-15.52) | 1.34(0.55-3.22) |
| Rather noisy-slightly | 2.39(1.50-3.81)*** | 2.68(0.81-8.95) | 2.39(1.44-3.96)** |
| Rather noisy-moderate | 2.83(1.76-4.55)*** | 1.91(0.55-6.67) | 3.13(1.87-5.24)*** |
| Rather noisy-significant | 2.66(1.68-4.22)*** | 2.79(0.84-9.29) | 2.70(1.63-4.45)*** |
| **Noise in the rest area-perceived noise impact** | |  |  |
| Quiet or moderate-no effect | Reference | Reference | Reference |
| Quiet or moderate-slightly | 2.32(1.57-3.45)*** | 1.90(0.75-4.82) | 2.45(1.59-3.79)*** |
| Quiet or moderate-moderate | 2.55(1.70-3.83)*** | 1.84(0.70-4.84) | 2.75(1.76-4.31)*** |
| Quiet or moderate-significant | 2.69(1.78-4.06)*** | 2.22(0.84-5.85) | 2.84(1.80-4.48)*** |
| Rather noisy-no effect | 9.44(1.38-64.51)* | - | 15.97(1.42-180.05)* |
| Rather noisy-slightly | 2.12(1.18-3.79)* | 1.43(0.40-5.13) | 2.36(1.22-4.57)* |
| Rather noisy-moderate | 2.71(1.59-4.63)*** | 1.63(0.47-5.69) | 3.15(1.74-5.74)*** |
| Rather noisy-significant | 2.60(1.69-4.00)*** | 2.03(0.74-5.56) | 2.76(1.71-4.44)*** |

Note: * indicated *P*<0.05; ** indicated *P*<0.01; ** indicated *P*<0.001. OR, odds ratio; CI, confidence interval.

All regression models were adjusted for age, household registration, marital status, education, smoking status, physical activity, dietary habits, shift work, monthly income, sleep duration, hypertension, dyslipidemia, diabetes mellitus or impaired glucose tolerance and BMI (continuous variable).
